# *Propionibacterium (Cutibacterium) granulosum* Extracellular DNase BmdE Targeting *Propionibacterium (Cutibacterium) acnes* Biofilm Matrix, a Novel Inter-Species Competition Mechanism

**DOI:** 10.3389/fcimb.2021.809792

**Published:** 2022-01-13

**Authors:** Vicky Bronnec, Hinnerk Eilers, Anika C. Jahns, Hélène Omer, Oleg A. Alexeyev

**Affiliations:** Department of Pathology, Medical Biosciences, Umeå University, Umeå, Sweden

**Keywords:** *Propionibacterium*, *Cutibacterium*, biofilm, matrix-degrading enzyme, extracellular nuclease, inter-species competition, acne vulgaris

## Abstract

Acne vulgaris is the most common dermatological disorder worldwide affecting more than 80% of adolescents and young adults with a global prevalence of 231 million cases in 2019. The involvement of the skin microbiome disbalance in the pathophysiology of acne is recognized, especially regarding the relative abundance and diversity of *Propionibacterium acnes* a well-known dominant human skin commensal. Biofilms, where bacteria are embedded into a protective polymeric extracellular matrix, are the most prevalent life style for microorganisms. *P. acnes* and its biofilm-forming ability is believed to be a contributing factor in the development of acne vulgaris, the persistence of the opportunistic pathogen and antibiotic therapy failures. Degradation of the extracellular matrix is one of the strategies used by bacteria to disperse the biofilm of competitors. In this study, we report the identification of an endogenous extracellular nuclease, BmdE, secreted by *Propionibacterium granulosum* able to degrade *P. acnes* biofilm both *in vivo* and *in vitro*. This, to our knowledge, may represent a novel competitive mechanism between two closely related species in the skin. Antibiotics targeting *P. acnes* have been the mainstay in acne treatment. Extensive and long-term use of antibiotics has led to the selection and spread of resistant bacteria. The extracellular DNase BmdE may represent a new bio-therapeutical strategy to combat *P. acnes* biofilm in acne vulgaris.

## Introduction

Biofilm formation is a surviving strategy contributing to the composition and long-term stability of the microbial populations (Rendueles and Ghigo, 2015; Flemming et al., 2016). Extracellular DNA (eDNA) is an important component of the matrix and its degradation is used by bacteria to modify biofilms (Flemming et al., 2016; Rumbaugh and Sauer, 2020). Within biofilms, secretion of nucleases has been described mostly as a strategy used by pathogens to evade the host immune system (Sharma et al., 2019). Whether endogenous matrix-degrading nucleases have any role in the setting of inter-species competition within biofilms is unknown.

*Propionibacterium* (*Cutibacterium*) acnes is a part of the normal skin microbiota dominating in the pilosebaceous unit (PSU), a hostile lipid-rich environment, low in oxygen and nutrients (Jahns et al., 2012; Jahns et al., 2013a). *P. acnes* ability to form biofilms is associated with the persistence of the pathogen and may also represent an adaptive strategy (Coenye et al., 2007; Dessinioti and Katsambas, 2017). The skin commensal *Propionibacterium granulosum*, while colonizing the same PSU as *P. acnes*, is commonly seen as a separate community (Jahns et al., 2015) implying a possible existence of a competitive mechanism. *P. acnes* biofilms are dominant in PSU (Jahns et al., 2012; Jahns et al., 2013a) and we hypothesized that a biofilm-degrading mechanism may be beneficial for *P. granulosum* colonization. We further speculated that this mechanism should be executed through a component secreted by *P. granulosum*.

In the present study we focused on identifying a possible antagonistic compound produced by *P. granulosum* facilitating colonization of PSU in the presence of *P. acnes*.

## Materials and Methods

### Bacterial Strains and Planktonic Culture Conditions

*P. acnes* KPA171202 and *P. granulosum* DSM 20700 (DSMZ, Braunschweig, Germany) were cultured under anaerobic conditions at 37°C and rotation 250 rpm as previously described (Bronnec and Alexeyev, 2021).

### *P. granulosum* DSM 20700 Genome Sequencing

Total DNA from *P. granulosum* was extracted as previously described (Bronnec and Alexeyev, 2021). Paired-end sequencing was performed using Illumina HiSeq2000 2x100 bp runs. Reads were mapped against the reference genome GCA_900186975.1 and checked using Kraken 2. Quality control was performed using FastQC v0.11.9. *De novo* assembly and annotation were performed using RAPT release v0.3.2 on the reads filtered with KrakenTools. Data were analyzed and edited using NCBI Genome Workbench software version 3.6.

### Biofilm Formation

Sessile bacteria were grown in brain heart infusion broth supplemented with 2 g/L of glucose (BHI_g_) either in a volume of 10 mL in T-25 cell culture flasks (Sarstedt AB, Helsingborg, Sweden) or 200 µL in a 96-well polystyrene culture plate with flat bottom (Sarstedt AB). Biofilms were kept for 6 days with medium changes every other day. Incubations were done under anaerobic conditions at 37°C.

### Cell‐Free Supernatant Preparation

To prepare cell‐free supernatant (CFS), 10 mL of bacterial culture were centrifuged 10 minutes at 3 500 g at room temperature. The supernatant was filtered through a 0.22 μm syringe filter (Sarstedt AB). CFS was fractionated according to the following molecular weights: < 30 kDa, < 50 kDa, > 30 kDa and > 50 kDa using a size exclusion centrifugation device (Amicon^®^ Ultra-15 Centrifugal Filter Units, Merck Chemicals and Life Science AB, Solna, Sweden) according to the manufacturer protocol.

### Effect of *P. granulosum* CFS on *P. acnes* Biofilm

The culture medium of a six-days old *P. acnes* biofilm was removed. The biofilm was washed with PBS 1X and incubated with *P. granulosum* CFS at 37°C for 24- and 48 hours. Controls included *P. acnes* biofilm incubated with either BHI_g_, *P. acnes* CFS or fresh planktonic cultures of *P. granulosum* or *P. acnes*. Effect of *P. granulosum* CFS on *P. acnes* biofilm was performed at least in triplicates with three biological replicates.

### Production of the Recombinant Protein BmdE

The sequence L860_000289 (**Table 1**), was amplified by PCR using the Phusion™ High-Fidelity DNA Polymerase (Fisher Scientific, Göteborg Sweden) with the forward primer 5’-GTACGAAGACTCCATGGGCCCAGCTCCCCACCC-3’ (with added NcoI restriction site) and reverse primer 5’-GTACAAGCTTCAGATGCCGGTGGCCG-3’(with added HindIII restriction site). The amplified sequence was inserted in frame into the pET HisZZ-TEV vector and the construct was confirmed by Sanger sequencing. The protein was overexpressed in *Escherichia coli* strain Rosetta (DE3) and purified thought Ni-NTA column followed by ion exchange and size-exclusion chromatography. Both His- an ZZ- tags were removed by enzymatic cleavage.

**Table 1:** *P. granulosum* genome data and L860_000289 main characteristics.

Genome	*Cutibacterium granulosum (formerly Propionibacterium granulosum)*			
StrainDSM 20700	BioProjectPRJNA203685		BioSampleSAMN02798018	AccessionJNBU02000000
**Gene**	**BmdE**			
**Locus tag** L860_000289	>JNBU02000015_1:33301-36099			
**Location** Complement 33301-36099	TCAGATGCCGGTGGCCGGCAGGGCCGGTAGATCGGCAGACCCTCCAGGTACGACGGCAGCCTGAGCGGCATCCCCCGACA			
**Contig** N°15	CGTCGTCAGTACCGGTTCCATGTGCGGATGCCGAAGCCCCCGCCGGTGTGGCGCTCGGAGTGCCGGGCGTCCCACCAGGC			
**Length** 2 799 bp	AGTGACGGAACCGGAGTCAGCGACGGAGCCGGGGTACCAGGCGTCACACTCGCAGCAGCACTGGGCTCGTCACTCACAGC			
	GCCACTCGGCGTGGCAGGCGACGCAGTGGGCGAAACAGACGGCTCACCACTGGGCGGTGTCGACACCGACGCGCTCGGCT			
	CGACGGACGAGGTTTCACTGGGCTGCGGGGTCGACGCGCCCGGGAACGTGATCCCCACCAGGGCGGGATCGTGGTCGGAG			
	GCAGCCCATGGTCCGCTCGTGTGGAAATCCGTCACATTGTAGTTGCGACGGGAGTACTGCATGGCGATGGACTCGTCGGC			
	ATTGATGTCCCACACCCCGGCACCACTGACCATCCTCATGGCCGCCGCGTTGGCGAAGATGTGGTCCAGGGAACCCAGCC			
	GTCCACCGTACTGGAAGGTCGACGCGCCAGGAGCGAGCTGCTCGACGACCTCGGTGTAGCCGGCGTCCTTGATCTTCATG			
	ACCGGATCCTCCATGCCGTAGGCGTTGAAGTCACCGAGCAGGAACACCGGCTCGCCGGCGAACTCCTTGGCGCTCCAGTC			
	GATCAGTGCCTGGGCCTGGACAGTACGTGAGGGGTTCGACTTGCCCTGACCGGTGCCGTCGTCCTCCCCGGAACCCTTCG			
	ACTTGAAGTGATTGGCGACGGCGACGAAGGAGCCCCTGACGTCCTTGGCCACGAACTTCTGGGCCAGTGGCTGACGGGCA			
	TTGGCGTAGGCATCATCCATGAGGATGAGCGATGGTCCCTCCGGGCGCACCGAGTCGAGTCGGTAGATGAACGCGGTACG			
	GATGACGTCCTCATGGGGCGGCACGACGGTCGGCGAGGCGACGTATCCCCAGACCTGGGAACCAGCAGCCTTGTTGAGTT			
	CGTCCACCAGATGGGCCAGGGCCTTGTCACGAGGCTGACCGGGCAGGTAGGTGATGGACGCGGAGTTCTCCACCTCCATG			
	AGCGCGACGACGTCGGCGTCCAAACCGTTGATGGCCGTGACGATCTTGGCCTGCTGGTCGTGGAATGCCTCTCGGGTGTA			
	GGCGCCACGCACCGTGCAGAAATTGGCGGTGACCGGCGTGCCGGTGCGATCCGTGTAGGCCTTGCAGCCGTCCTCGTCCT			
	TGCCCAGGTCGGTGAAGTAGTTGAGCACGTTGAAGCTGGCGAGCTTGAGATCGGAGTCGAAACTCGGGGCCTTGGCCGGA			
	CGATCGTTGGTGGTGGTGATCGGACTGTGGGGCGACTGGGCGCCCGAGACCATGCCAGTGGGCTGGAAGTTCCACCCGAA			
	CCGGTAGTCCATGATCACCGGTCTGGTGAAGCCCACGTGGGAGCCGGTACGCATCGGTGTGTCCTGGGACAGGTACGGCA			
	GCGGGGAGTTCTTGGCCTCGTCGTTGGTCTGATAGTTCCAGCTCGCACCGTCGTCCAGATTGATGGATCGGGCACGGTTC			
	TCGTCCTCGACCTTCTTGGCATCCTCACCGGGACGGGCCACCTCGGTACCTTGGTACAGCGGCTCGGTGCCGAAGGCGAG			
	GCCAACGGTGCCGAACTGGTTGAGCTGGTAATTGTTAGAGATGGTGTAGGTGCCCTGTGGTTCCACGAGCATGGACTCAT			
	ACGCCTCACGCTGCGCATCGGTGCGCGGCAGGGTTGCGAGTTTCACCGGCTTGACGTCGGCACAGCCCTTGGTCTCGGTG			
	ACGCTCGTCCCATTGATCTCGGTGAGACCGTTGAACTCCGAGACCTCACCGGAGACGACGACGCACTGACCTGCGGTGTA			
	GGCGCTGGCGGCCTTGGCGGAGTGGACGAAGATGCCGTCGGAGGCATCGGTCGGGGTCTTGGCCACACCACCAGAGCCGG			
	GAGTCTGGATGTAGAGGCCATTGAACCCGCCCTTGGGGTAGACGGCGGTGACGACGCCCGTGGTGGTCACGACCTTGCCG			
	ACCATCGGGCTGGTGTCGGTGGTGCCCTGGATGTCGGCGATGCTCGTCTGGCCAGTCGGGGTCTCGGAGGCAGTGGGGGT			
	CGCAGAACCGCTCGGAGTGGGGCTGGCCGTCTGGTCAGGAGTGCCTGGCGTGCTGGGAGCCGGTGTGCTGGCGGAGTTCA			
	TCGGGCTGGGAGTGCCGATGACGAAGTCCGCGGAGTTGTCGTCGGTGTCCTCGAGGCCACCCCGCTGGGCCGAGGTGGTG			
	TTCGACAGTGCCTTGGTCGGTTTTCCCTCGGCCTTGGCGGCGGCACCGTAGCCGACGAGGTCGACGACCGTGCCGTCCGG			
	GCCGACCAGCTGCACCGATCCCTTGGTGCCACTCATGTTGGCACCGCAGGTGGCATCCGGGGTGGGCAGTGCCTGGGTAC			
	CACCGGCTCCCTTGGCCTGCTGGACGAGGAAGTGACCGCCGGGACCGACCGACCCGCTCAACGTGCACCTGTTTCCGCGA			
	TTGCCGGTGGCTCCGTAGTACTCCACGACGTACCCGTCCAGCGAGATCGTCTCGGAGGTGGGATTGACGAGCTCGACGAA			
	GTCGTGGGTGAGGGTGGCTCCACTGTTGCCACCGCCACCGTAGACCTCATTGATGAGTGGGTGGGGAGCTGGGCCGGCAT			
	TGGCACTTGGCACGAGCAGACCGGCACATGCGAGGGCCGAGGTCGTCACCGCGGCGATACCGGTCAGCACCCGGCGCAT			
**Protein**	**BmdE: Biofilm Matrix Degrading Enzyme**			
**Description** Extracellular DNase BmdE; Biofilm matrix degrading eDNAse	>KAG9060303.1_BmdE_[Cutibacterium_granulosum_DSM_20700]			
**Protein_id:** KAG9060303.1	MRRVLTGIAAVTTSALACAGLLVPSANAGPAPHPLINEVYGGGGNSGATLTHDFVELVNPTSETISLDGYVVEYYGATGN			
**Length / Size** 932 aa / 96 kDa	RGNRCTLSGSVGPGGHFLVQQAKGAGGTQALPTPDATCGANMSGTKGSVQLVGPDGTVVDLVGYGAAAKAEGKPTKALSN			
	TTSAQRGGLEDTDDNSADFVIGTPSPMNSASTPAPSTPGTPDQTASPTPSGSATPTASETPTGQTSIADIQGTTDTSPMV			
	GKVVTTTGVVTAVYPKGGFNGLYIQTPGSGGVAKTPTDASDGIFVHSAKAASAYTAGQCVVVSGEVSEFNGLTEINGTSV			
	TETKGCADVKPVKLATLPRTDAQREAYESMLVEPQGTYTISNNYQLNQFGTVGLAFGTEPLYQGTEVARPGEDAKKVEDE			
	NRARSINLDDGASWNYQTNDEAKNSPLPYLSQDTPMRTGSHVGFTRPVIMDYRFGWNFQPTGMVSGAQSPHSPITTTNDR			
	PAKAPSFDSDLKLASFNVLNYFTDLGKDEDGCKAYTDRTGTPVTANFCTVRGAYTREAFHDQQAKIVTAINGLDADVVAL			
	MEVENSASITYLPGQPRDKALAHLVDELNKAAGSQVWGYVASPTVVPPHEDVIRTAFIYRLDSVRPEGPSLILMDDAYAN			
	ARQPLAQKFVAKDVRGSFVAVANHFKSKGSGEDDGTGQGKSNPSRTVQAQALIDWSAKEFAGEPVFLLGDFNAYGMEDPV			
	MKIKDAGYTEVVEQLAPGASTFQYGGRLGSLDHIFANAAAMRMVSGAGVWDINADESIAMQYSRRNYNVTDFHTSGPWAA			
	SDHDPALVGITFPGASTPQPSETSSVEPSASVSTPPSGEPSVSPTASPATPSGAVSDEPSAAASVTPGTPAPSLTPVPSL			
	PGGTPGTPSATPAGASASAHGTGTDDVSGDAAQAAVVPGGSADLPALPATGI			
**BLAST**	**Nucleotide sequence analysis of L860_000289**			
(analysis performed on the available genomes at the date 2020.06.01)		**Coverage**	**Identity**	**Similarities**
	***P. granulosum***	99 %	94 %	95 %
	***P. avidum***	89 %	60 %	71 %
	***P. acnes***		No significant similarity found	

### Qualitative Evaluation of *P. acnes* Biofilm Dispersion

*P. granulosum* CFS containing protein fractions with different molecular weights were tested*. P. acnes* biofilm grown in 96-well plate was treated with 200 µL of *P. granulosum* CSF fractions for 24 hours at 37°C. Dispersal activity of the recombinant protein r-BmdE was evaluated with DNase I (Sigma-Aldrich Sweden AB, Stockholm, Sweden) as a positive control. An equal molar concentration of both enzymes (1 x 10^-11^ mol/mL) was tested in 10 mL. Biofilms were incubated for 24 hours. PBS 1X and r-BmdE buffer (50 mM NaP, 50 mM NaCl, 10 % glycerol, 2 mM β-Mercaptoethanol) were used as a negative control. The dispersal effect was visually evaluated. The experiment was performed in triplicate.

### DNase Activity Assays

The DNase activity of *P. granulosum* CFS was evaluated as follows. The largest fraction > 50 kDa was concentrated 50 times using ultrafiltration devices according to the manufacturer protocol (Amicon^®^). Several volumes (1 µL, 3 µL and 5 µL) of this faction were incubated with 500 ng of *P. acnes* DNA for two hours at 37°C. *P. acnes* DNA was extracted as previously described (Bronnec and Alexeyev, 2021). As controls, reaction mix were supplemented with 3 mM MgCl_2_ and 10 mM EDTA. Samples were run on a 1 % (w/v) agarose gel containing GelGreen^®^ Nucleic Acid Stain (VWR) to visualize DNA degradation.

A semi-quantitative analysis of biofilm dispersal activity was performed with crystal violet assay. *P. acnes* biofilms grown in a 96-well plate were washed with PBS 1X and treated with 200 µL of serial dilutions of enzymes ranging from 500 pmol to 3 x 10^-9^ pmol per well. After two hours incubation at 37°C crystal violet assay was performed as previously described (Peeters et al., 2008) with some modifications. Biofilms were stained with 0.1% (w/v) of crystal violet (Fisher Scientific). The solubilization was performed with 200 µL of a solution composed of 80% ethanol and 20% acetone (v/v). The absorbance was read at 560 nm. This experiment was performed with at least three technical and three biological replicates.

The DNase activity of the recombinant protein on *P. acnes* DNA was tested in the same way using different quantity of r-BmdE ranging from 5 µg to 0.2 µg per µg of DNA and incubated for 2 hours at 37°C. A six-days old preformed biofilm of *P. acnes* was incubated with 0.001 mg/mL of r-BmdE. The dispersal activity was visually observed after 24 hours of incubation. r-BmdE activity was also tested after pre-incubation of the enzyme with 10 % and 5 % (v/v) Intralipid^®^ solution (Fresenius Kabi, Uppsala, Sweden) for 2 hours at room temperature followed by incubation with 1 µg of *P. acnes* DNA for 2 hours at 37°C. Lipids were removed after two successive centrifugations at 13 000 rpm for 10 min at 4°C.

### *In Vivo P. acnes* Biofilm Dispersion With r-BmdE

The *in vivo* model of *P. acnes* biofilm in *Drosophila melanogaster* (Bronnec and Alexeyev, 2021) has been used to evaluate the dispersal activity of BmdE. Briefly, the feeding infection method has been used to develop a mature biofilm of *P. acnes* in the fruit fly gut. The biofilm was treated for three and five days with 0.65 nmol of r-BmdE. The fruit fly diet has been modified and supplemented at 11 % with Intralipid^®^ solution (Bronnec and Alexeyev, 2021). A treatment with BHIg was used as a control. To evaluate the biofilm dispersion, formalin fixed, paraffin embedded flies were sectioned and observed with a light microscope. Biofilm positive sections were previously defined as sections containing large microbial structures attached to the gut wall in the abdominal part of the fly (Bronnec and Alexeyev, 2021). Other sections are considered as biofilm negative and correspond to a partial or a totally dispersed biofilm. These criteria were followed to evaluate *P. acnes* biofilm dispersion with r-BmdE.

To evaluate if *P. acnes* viability was affected by r-BmdE treatment, we used a live/dead staining kit as well as bacterial culture. First, *P. acnes* planktonic cultures were incubated with 50 %, 33 %, 10 % and 1% of r-BmdE for 30 minutes and 16 hours at 37°C, with r-BmdE buffer and BHIg as controls. LIVE/DEAD™ BacLight™ Bacterial Viability Kit (Thermo Fisher Scientific, Upsala, Sweden) was used according to the manufacturer protocol. Then, fruit flies excretions containing *P. acnes* released from the biofilm were collected after the treatment. The Whatman filter of the fruit fly vial was resuspended in 100 µL of buffered peptone water and the suspension was plated and incubated in anaerobically at 37°C.

## Results

**Figure 1:**
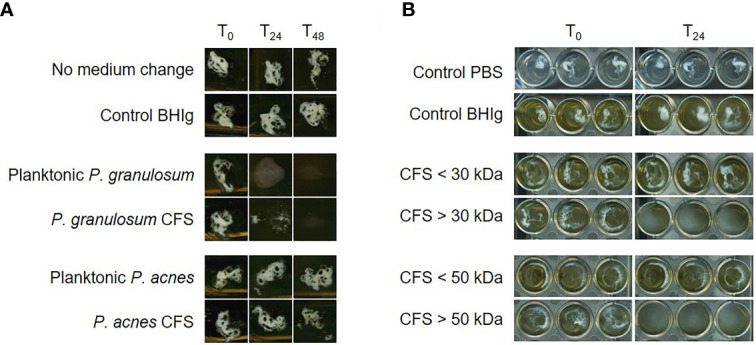
*P. acnes* biofilm dispersion by *P. granulosum* cell‐free supernatant (CFS). **(A)** Dispersal of 6 days old *P. acnes* biofilms in flask after 24 and 48 hours CFS treatment. **(B)** *P. acnes* biofilm dispersion in 96-well plate after 24 hours treatment with different *P. granulosum* CFS fractions.

A co-culture of *P. granulosum* as well as cell‐free supernatant (CFS) of *P. granulosum* with a six-days old *P. acnes* biofilm resulted in total dispersion of the biofilm after 48 hours (**Figure 1A**). Size-fractionation of the CFS further showed that *P. acnes* biofilm is degraded by a fraction size exceeding 50 kDa (**Figure 1B**). The absence of biofilm dispersion using *P. acnes* CFS indicated that the secreted protein is not conserved within *P. acnes* strains.

We further sequenced the *P. granulosum* DSM 20700 genome. This Whole Genome Shotgun project has been deposited at DDBJ/ENA/GenBank under the accession JNBU00000000. The version described in this paper is version JNBU02000000. A bioinformatical analysis of the genome was performed using the following criteria: (i) a size ≥ 50 kDa, (ii) prediction of secretion-tag from amino acid sequence, (iii) a gene annotation keywords search “extracellular protein”, “secreted protein” or “uncharacterized protein” and (iv) sequence not conserved among *P. acnes* genomes. The first-choice candidate after the *in-silico* search corresponded to an uncharacterized extracellular nuclease (L860_000289) encoding for a 939 aa protein (**Table 1**). BLAST sequence analysis showed a high conservation of the sequence encoding this protein in *P. granulosum* strains (**Table 1**).

**Figure 2:**
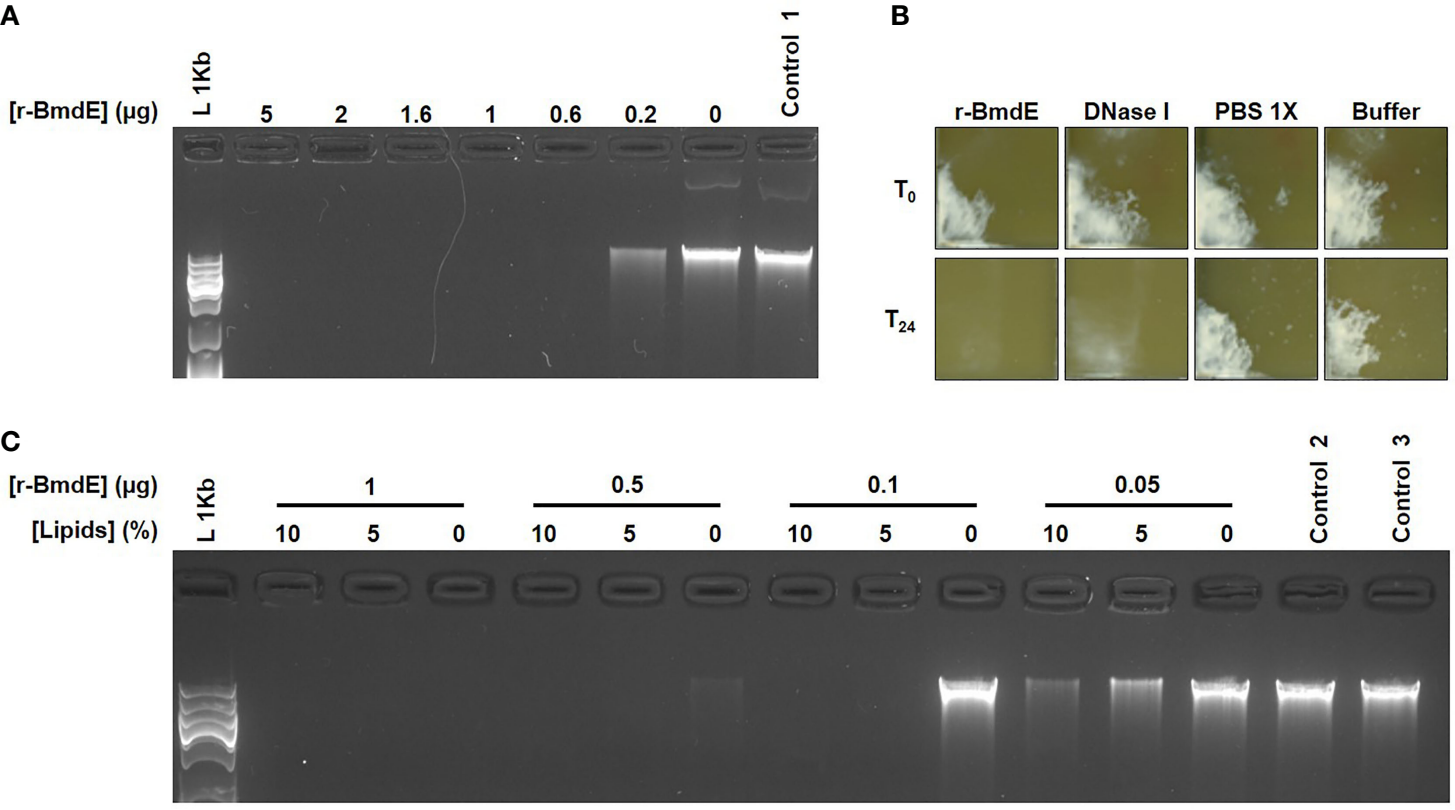
*In vitro* deoxyribonuclease activity of r-BmdE. **(A)** *P. acnes* DNA treated with r-BmdE. Control 1 consisted of r-BmdE buffer at the highest volume. **(B)** Mature biofilm of *P. acnes* incubated r-BmdE. An equal molar ratio of DNase I (0.0003 mg/mL) was used as a positive control and 10 µL of PBS 1X and r-BmdE buffer were used as negative controls. **(C)** r-BmdE activity in lipids. Controls 2 and 3, consisted of r-BmdE buffer and water respectively.

We further demonstrated that the CFS fraction > 50 kDa exhibited a DNase activity by degrading *P. acnes* DNA. The activity was enhanced with cations and inhibited with EDTA. We named this extracellular nuclease as BmdE (biofilm matrix degrading enzyme, eDNase BmdE) according to International Protein Nomenclature Guidelines from NCBI (NCBI, Last updated: 02-MAR-2020) (**Table 1**). Recombinant BmdE was produced and showed the ability to degrade *P. acnes* DNA (**Figure 2A**) as well as preformed biofilm of *P. acnes in vitro* (**Figure 2B**). A quantitative biofilm dispersion analysis was performed in 96-well plate with serial dilutions of r-BmdE (500 pmol to 3 x 10^-9^ pmol of enzyme/well). A quantity of 0.03 ng of r-BmdE was the lowest one used to disperse a mature biofilm of *P. acnes in vitro* compared to 0.25 ng for the control with DNase I. In the conditions tested, no lethal effect of r-BmdE was observed using the viability assay kit. Pre-incubation of r-BmdE in a lipid-rich environment showed enhanced DNA hydrolysis (**Figure 2C**).

We recently reported a successful development of an *in vivo* model of *P. acnes* biofilm in fruit fly on a lipid rich diet at an attempt to mimic a lipid rich PSU environment (Bronnec and Alexeyev, 2021). A 3- and 5 day *in vivo* treatment of *P. acnes* biofilm with r-BmdE resulted in biofilm degradation in both lipids and non-lipids environment (**Table 2**).

**Table 2:** *In vivo* effect of r-BmdE treatment on *P. acnes* biofilm.

Treatment	Supplement	3 Day Treatment				5 Day Treatment			
		Flies	Sections			Flies	Sections		
		Number	n (%)		*p*	Number	n (%)		*p*
		Total	Positive	Negative		Total	Positive	Negative	
BHI_g_	No	9	162 (80)	41 (20)	< 0.0001 *^a^*	9	147 (68)	24 (32)	< 0.0001 *^a^*
r-BmdE		10	44 (25)	135 (75)		7	10 (6)	170 (94)	
BHI_g_	Intralipid^®^	9	85 (51)	82 (49)	< 0.0001 *^a^*	6	75 (54)	64 (46)	< 0.0001 *^a^*
r-BmdE		5	8 (11)	66 (89)		4	16 (16)	81 (84)	

Values are presented as number (percentage).

BHI_g_, brain heart infusion broth supplemented with 2 g/L of glucose.

r-BmdE, Recombinant extracellular nuclease BmdE (biofilm matrix degrading enzyme).

a Effect of enzyme treatment was compared to the control, p values were calculated using the two-tailed Fisher’s exact test.

Results show that r-BmdE treatment does not impact *P. acnes* viability since the bacteria released from biofilm could be regrown on agar plate supporting results obtained with the viability assay kit.

## Discussion

Our results indicate that *P. granulosum* is able to secret BmdE, an eDNase disrupting *P. acnes* biofilm. Highly conserved within *P. granulosum* strains BmdE can represent an antagonistic compound facilitating colonization in the presence of *P. acnes*. *In vitro*, DNase activity of r-BmdE appears to be enhanced in lipids solution and retain activity *in vivo* in *D. melanogaster* on lipid-rich diet. This is in line with the observation that *P. granulosum* producing BmdE, naturally inhabits the PSU, a sebum/lipids-rich environment.

During bacterial colonization, competition for nutrients or adhesion sites require that one of the interacting microorganisms inhibits the development of other species through the production of antagonistic compounds. In bacterial biofilms, eDNA facilitates adhesion, helps to maintain the structural integrity of the biofilm. It is assumed that eDNA contributes to antimicrobial resistance and protects from the host immune system (Okshevsky and Meyer, 2015; Flemming et al., 2016). Microorganisms are also able to secrete enzymes to modulate their own biofilm. Based on this mechanism, several strategies are available to change the matrix stability using enzymes targeting eDNA at different stages of the biofilm life cycle (Tetz et al., 2009). The commensal *Staphylococcus epidermidis* has been reported to secret a protease interrupting the release of eDNA which consequently inhibits biofilm formation and nasal colonization by *Staphylococcus aureus* (Iwase et al., 2010; Chen et al., 2013; Stubbendieck and Straight, 2016). Our data suggest that the ability to produce a DNase can represent a novel competitive mechanism targeting biofilm within the same genus. This mechanism can probably explain our earlier observation that *P. acnes* and *P. granulosum* form separate communities while colonizing the same PSU (Jahns et al., 2015).

*P. acnes* biofilm formation is believed to be an important pathogenic mechanism in acne vulgaris (Jahns et al., 2012; Jahns et al., 2013b). Biofilms are thought to be able to directly block hair follicles, contribute to antibiotic resistance and inflammation. The use of BmdE in acne treatment for controlling the colonization and biofilm formation by *P. acnes* might be an innovative strategy. The use of this non-biocidal molecule, fully functional in its natural lipid-rich environment both *in vitro* and *in vivo*, may alter the biofilm structure by degrading eDNA and lead to the release of planktonic *P. acnes* susceptible to antibiotics. The usage of nucleases such as the recombinant human DNase is an effective and safe approach already described for *Pseudomonas aeruginosa* biofilm treatment in cystic fibrosis infection (Patel et al., 2020; Yang and Montgomery, 2021). Using enzymes that can weaken or destroy the biofilm structure can represent a new strategy to combat bacterial biofilms in acne vulgaris.

## Data Availability Statement

The datasets presented in this study can be found in online repositories. The names of the repository/repositories and accession number(s) can be found in the article/supplementary material.

## Author Contributions

VB conceived, designed, and performed experiments, and wrote the manuscript. HE, AJ, and HO conceived, designed, and performed experiments. OA conceived and designed experiments, and wrote the manuscript. All authors contributed to the article and approved the submitted version.

## Funding

Financial support was provided through a regional agreement between Umeå University and Västerbotten County Council on cooperation in the field of Medicine, Odontology and Health (ALF), the Kempe Foundation and Umeå Biotech Incubator.

## Conflict of Interest

Based on these findings an international patent application has been filed (patent #WO2020190203A1: “New compositions and methods for the treatment of acne vulgaris”, 19 March 2020) by Vakona AB where OA is a part owner. This does not alter the authors’ adherence to all the Frontiers Journals policies on sharing data and materials.

The remaining authors declare that the research was conducted in the absence of any commercial or financial relationships that could be construed as a potential conflict of interest.

## Publisher’s Note

All claims expressed in this article are solely those of the authors and do not necessarily represent those of their affiliated organizations, or those of the publisher, the editors and the reviewers. Any product that may be evaluated in this article, or claim that may be made by its manufacturer, is not guaranteed or endorsed by the publisher.
